# Recommendations for a practical implementation of circulating tumor DNA mutation testing in metastatic non-small-cell lung cancer

**DOI:** 10.1016/j.esmoop.2022.100399

**Published:** 2022-02-21

**Authors:** E. Heitzer, D. van den Broek, M.G. Denis, P. Hofman, M. Hubank, F. Mouliere, L. Paz-Ares, E. Schuuring, H. Sültmann, G. Vainer, E. Verstraaten, L. de Visser, D. Cortinovis

**Affiliations:** 1 Institute of Human Genetics, Diagnostic and Research Center for Molecular Biomedicine, Medical University of Graz, Graz, Austria; 2 Antoni van Leeuwenhoek-Netherlands Cancer Institute, Amsterdam, The Netherlands; 3 Department of Biochemistry and Molecular Biology, Nantes University Hospital, Nantes, France; 4 Laboratory of Clinical and Experimental Pathology, FHU OncoAge, Biobank BB-0033-00025, University Côte d’Azur, Nice, France; 5 The Royal Marsden NHS Foundation Trust, London, UK; 6 Department of Pathology, Amsterdam UMC, Vrije Universiteit Amsterdam, Cancer Center Amsterdam, Amsterdam, The Netherlands; 7 Department of Medical Oncology, Hospital Universitario 12 de Octubre, H12o-CNIO Lung Cancer Unit, Universidad Complutense & CIBERONC, Madrid, Spain; 8 Department of Pathology and Medical Biology, University of Groningen, University Medical Center Groningen, Groningen, The Netherlands; 9 Division of Cancer Genome Research, German Cancer Research Center (DKFZ), German Center for Lung Disease (DZL) and German Cancer Consortium (DKTK), Heidelberg, Germany; 10 Department of Pathology, Hadassah Medical Center, Faculty of Medicine, Hebrew University of Jerusalem, Jerusalem, Israel; 11 Roche Diagnostics, Almere, The Netherlands; 12 Roche Diagnostics International, Rotkreuz, Switzerland; 13 SC Medical Oncology, Azienda Socio Sanitaria Territoriale (ASST) H S Gerardo Monza, Monza, Italy

**Keywords:** ctDNA mutation testing, non-small-cell lung cancer, precision oncology, patient scenario

## Abstract

**Background:**

Liquid biopsy (LB) is a rapidly evolving diagnostic tool for precision oncology that has recently found its way into routine practice as an adjunct to tissue biopsy (TB). The concept of LB refers to any tumor-derived material, such as circulating tumor DNA (ctDNA) or circulating tumor cells that are detectable in blood. An LB is not limited to the blood and may include other fluids such as cerebrospinal fluid, pleural effusion, and urine, among others.

**Patients and methods:**

The objective of this paper, devised by international experts from various disciplines, is to review current challenges as well as state-of-the-art applications of ctDNA mutation testing in metastatic non-small-cell lung cancer (NSCLC). We consider pragmatic scenarios for the use of ctDNA from blood plasma to identify actionable targets for therapy selection in NSCLCs.

**Results:**

Clinical scenarios where ctDNA mutation testing may be implemented in clinical practice include complementary tissue and LB testing to provide the full picture of patients’ actual predictive profiles to identify resistance mechanism (i.e. secondary mutations), and ctDNA mutation testing to assist when a patient has a discordant clinical history and is suspected of showing intertumor or intratumor heterogeneity. ctDNA mutation testing may provide interesting insights into possible targets that may have been missed on the TB. Complementary ctDNA LB testing also provides an option if the tumor location is hard to biopsy or if an insufficient sample was taken. These clinical use cases highlight practical scenarios where ctDNA LB may be considered as a complementary tool to TB analysis.

**Conclusions:**

Proper implementation of ctDNA LB testing in routine clinical practice is envisioned in the near future. As the clinical evidence of utility expands, the use of LB alongside tissue sample analysis may occur in the patient cases detailed here.

## Highlights

• LB is a rapidly evolving diagnostic tool that may be an adjunct or an alternative to TB in clinic.
• Pragmatic scenarios for ctDNA mutation testing to identify actionable targets in NSCLC are explored.
• ctDNA mutation testing may identify resistance mechanisms, discordant clinical history, and intertumor/intratumor heterogeneity.
• ctDNA mutation testing may be useful if the tumor location is hard to biopsy or if an insufficient tumor sample was taken.
• Proper implementation of ctDNA mutation testing in routine clinical practice is envisioned in the near future.

## Introduction

Liquid biopsy (LB) is a rapidly evolving diagnostic tool for precision oncology that has recently found its way into routine practice as an adjunct or an alternative to tissue biopsy (TB). The concept of LB refers to the analysis of any tumor-derived material detectable in the blood and other bodily fluids, such as circulating tumor DNA (ctDNA) or circulating tumor cells.^1^

Although TB is the gold-standard diagnostic method used to obtain or to confirm the diagnosis of cancer, to define the histological subtype, or to identify actionable targets, molecular profiling from ctDNA in patients with non-small-cell lung cancer (NSCLC) has several advantages, including its minimally invasive nature, a continuous monitoring of genetic alterations, and a better reflection of intratumor and intertumor heterogeneity.^1^, ^2^, ^3^, ^4^, ^5^

The National Comprehensive Cancer Network (NCCN) 2021 and European Medicines Agency guidelines recommend that when there is insufficient tissue to allow for testing for clinically relevant predictive mutations and rearrangements (e.g. *EGFR*), repeat biopsy and/or plasma testing should be performed.^6^^,^^7^ ctDNA LB mutation testing may reduce the cost of patient care as well as the turnaround time from sampling to the test results; however, it is important to consider that testing serial samples can increase overall costs.^1^^,^^5^^,^^8^, ^9^, ^10^

In 2018, a multidisciplinary panel of experts from the International Association for the Study of Lung Cancer (IASLC) concluded that ‘LB approaches have significant potential to improve patient care, and immediate implementation in the clinic is justified in a number of therapeutic settings relevant to NSCLC’.^11^ Yet, ctDNA LB mutation testing has a number of limitations, including a higher risk of producing noninformative results (e.g. artifacts, mutations that are not currently actionable), and the need for increased levels of sensitivity and specificity to detect ctDNA (as ctDNA levels can be low in some patients, notably in those with nonshedding tumors or cases of brain metastases) and for improved concordance between tests.^4^^,^^12^^,^^13^

Despite a continuously growing body of knowledge of ctDNA biology, alongside many advances in the technology used for testing, uptake into clinical daily practice still represents only a small proportion of cases. There are currently few approved tests for the use of ctDNA LB in routine clinical practice in NSCLC (Table 1).

**Table 1:** Examples of CE-IVD- and FDA-approved ctDNA LB mutation assays in NSCLC

Test	Technology	Approval	Biomarker
cobas *EGFR* test v2 (Roche)^100^^,^^101^	Real-time PCR	FDA/CE-IVD	*EGFR*
therascreen® mutation kits (QIAGEN)^101^	Real-time PCR	FDA/CE-IVD	*EGFR*
ct*EGFR* Mutation Detection Kit (EntroGen, Inc.)^102^	Real-time PCR	CE-IVD	*EGFR*
Super-ARMS® *EGFR* Mutation Test (Amoy Diagnostics Co.)^103^	Real-time PCR	CE-IVD	*EGFR*
FoundationOne® Liquid CDx assay (Foundation Medicine)^104^^,^^105^	NGS: hybridization-based capture sequencing (324 genes)	FDA/CE-IVD	*ALK*, *EGFR*, *NTRK2*, *NTRK3*, TMB, MSI
Guardant 360® CDx assay (Guardant Health)^106^	NGS: hybridization-based capture sequencing (55 FDA-approved genes, 74 genes for RUO)	FDA/CE-IVD	*ALK*, *EGFR*, *KRAS*

*ALK*, anaplastic lymphoma kinase; CE-IVD, CE *in vitro* diagnostic; ctDNA, circulating tumor DNA; *EGFR*, epidermal growth factor receptor; FDA, The United States Food and Drug Administration; *KRAS*, Kirsten rat sarcoma virus; LB, liquid biopsy; MSI, microsatellite instability; NSCLC, non-small-cell lung cancer; NGS, next-generation sequencing; *NTRK2*, neurotrophic receptor tyrosine kinase 2; *NTRK3*, neurotrophic receptor tyrosine kinase 3; PCR, polymerase chain reaction; RUO, research use only; TMB, tumor molecular burden.

The objective of this review is to consider pragmatic scenarios for the use of ctDNA from blood plasma to identify actionable targets for therapy selection in metastatic NSCLC.

## Methodological considerations for ctDNA profiling

The high concordance rates of 91%-95% for relevant predictive markers between TB and LB allow exploration into the clinical applications of LB across the patient journey.^14^, ^15^, ^16^, ^17^, ^18^, ^19^, ^20^

**Figure 1:**
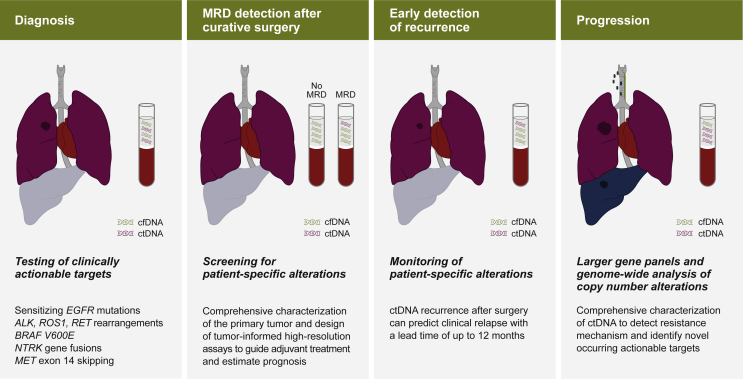
**Different clinical scenarios may require different analysis strategies.**^4^^,^^6^^,^^28^^,^^82^^,^^84^, ^85^, ^86^, ^87^ While in some cases hotspot testing might already reveal predictive information, in other scenarios such as MRD testing and identification of recurrences only the development of patient-specific, tumor-informed assays can achieve the necessary sensitivity. In late stages larger panels for *de novo* mutations or shallow whole-genome sequencing to identify novel actionable target is preferable. cfDNA, circulating free DNA; ctDNA, circulating tumor DNA; MRD, minimal residual disease.

Currently applied LB approaches include both polymerase chain reaction (PCR)-based methods and next-generation sequencing (NGS) technologies interrogating single genes, hotspot mutations, or large gene panels (Supplementary Table S1, available at https://doi.org/10.1016/j.esmoop.2022.100399). At present, consensus is needed on which methodologies may provide the best answers at various stages of the patient journey (Figure 1).^4^^,^^11^^,^^21^^,^^22^ PCR-based approaches such as digital droplet PCR or BEAMing (beads, emulsion, amplification, magnetics) are associated with higher sensitivity.^23^^,^^24^ However, the implementation of unique molecular identifiers, that help to correct errors and/or artifacts and quantitative biases, has led to a significant reduction of false-positive variant calls and to an increased sensitivity of variant detection for NGS techniques.^22^^,^^25^ While PCR-based approaches to analyzing well-characterized mutations in clinically relevant genes such as *EGFR* are most commonly used, larger NGS gene panels could be beneficial in specific clinical scenarios.^26^ Moreover, if a panel of genes are interrogated and one tumor variant is identified with a high variant allele frequency (VAF) that hints at a high tumor fraction, the absence of another clinically relevant mutation in this sample can indicate a true negative result.^27^

At the time of diagnosis, testing for clinically actionable and well-characterized alterations might already reveal predictive information (Figure 1). The NCCN Guidelines for NSCLC (version 7.2021)^6^^,^^28^ recommend molecular testing of sensitizing *EGFR* mutations; the *BRAF* V600E mutation; rearrangements involving *ALK*, *ROS1*, or *RET*; and exon 14 skipping of the *MET* gene. In later stages or during treatment, large panels for *de novo* mutation calling or even genome-wide approaches for copy number detection to detect resistance mechanism or to identify novel actionable targets are preferable. In particular, tyrosine kinase inhibitors (TKIs) and other targeted therapies can target a broad range of molecular alterations resulting in specific gene fusions or altered expression levels and are associated with multiple mechanisms of resistance that can be better captured by larger panels.^26^ In other scenarios, such as in a minimal residual disease (MRD) testing or early identification of relapses, only patient-specific assays based on the molecular profile of the tumor can achieve the required sensitivity. Most likely the described patient scenarios will not be covered by one ctDNA assay but many.

Ideally, combining liquid and tissue testing captures heterogeneity and can be highly sensitive without compromising specificity; however, this may be biased by the location of the tumor.^29^ Cost-effectiveness studies are needed to determine the benefit of this approach for patient outcomes versus the higher costs of combined testing.^30^

## Technical evaluation and validation of ctDNA workflows

**Figure 2:**
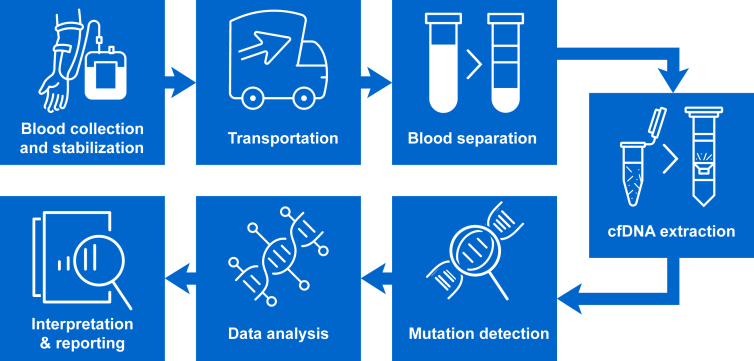
**Steps that need to be validated for ctDNA liquid biopsy workflows.**^11^^,^^31^, ^32^, ^33^, ^34^^,^^107^ Well-validated workflows are required for routine use as multiple steps are involved in such workflows. Although for some parts there is a consensus in the field (e.g. the use of preservatives or a double-spin protocol for plasma extraction), there are no guidelines on the timing of blood draw or which assay to use for which clinical application. Moreover, guidelines for data analysis, interpretation, and reporting are needed. *ALK*, anaplastic lymphoma kinase; CEA, carcinoembryonic antigen; CT, computed tomography; ctDNA, circulating tumor DNA; *EGFR*, epidermal growth factor receptor; IHC, immunohistochemistry; TKI, tyrosine kinase inhibitor; NGS, next-generation sequencing; NSCLC, non-small-cell lung cancer; PD-L1, programmed death-ligand 1.

Regardless of the platform, well-established and validated ctDNA LB workflows are essential for routine use.^31^, ^32^, ^33^, ^34^ ctDNA LB workflows include many steps that need to be validated^31^, ^32^, ^33^, ^34^ (Figure 2).

The preanalytical steps of ctDNA mutation testing can be impacted by many factors^13^ (Table 2). For analytical procedures, performance assessment should be done according to European Medicines Agency recommendations and International Organization for Standardization (ISO) standards to obtain reliable and comparable results.^35^^,^^36^

**Table 2:** Key preanalytical considerations for ctDNA mutation testing in NSCLC

Key preanalytical consideration	
Timing of blood draw^11^^,^^107^	• IASLC recommendations state the maximum time from blood withdrawal to plasma processing and storage should be 2 h for EDTA tubes and 3 days for preservative tubes at room temperature • Blood volume collected is ideally 2 × 10 ml; however, there is no standardized volume reported • Blood should be processed to plasma within 2 h of draw; alternatively, use of stabilization collection tubes containing fixatives should be considered to allow blood processing at a later time
Storage of cell-free plasma^11^^,^^107^	• Processed cell-free plasma should be stored at −20°C or −80°C (on dry ice for shipping), with long-term stability of DNA in plasma best demonstrated at −80°C
Sampling tube considerations^11^^,^^108^^,^^109^	• Blood should be collected in appropriate tubes to prevent coagulation • Preservative cfDNA Streck BCT tubes are designed to maintain quality of small DNA fragments for multiple days at room temperature. The manufacturer reports cfDNA stability of up to 14 days if stored appropriately, which allows convenient shipping to analysis laboratories • Yield remains stable in PAXgene ccfDNA, Streck BCT, and Roche cfDNA tubes for up to 48 h at room temperature (20-24°C) • EDTA tubes require processing on-site • Storage in EDTA tubes for between 4 and 48 h showed a small, but significant, increase in plasma cfDNA yields • For genomic analyses, EDTA tubes showed good results if stored for a maximum of 4 h at room temperature or for up to 24 h when stored at 4°C
Plasma and cfDNA-isolation protocols^11^^,^^107^^,^^109^	• A ‘double-spin’ plasma isolation procedure is highly recommended • To avoid loss of isolated cfDNA, samples should be stored in tubes with a low DNA-adsorption quality • Repeated freeze–thaw cycles should be avoided where possible • For maximal yield a method tailored for selective extraction of small cfDNA fragments (<166 bp) is recommended • Alternatively, size selection for short DNA fragments can be performed • Workflow for LB requires much more strict conditions compared with TB to prevent cross contamination
Quality assessment methods for cfDNA integrity^80^^,^^110^^,^^111^	• Fragmentation analysis (e.g. TapeStation) • Various size-based ddPCR fragments

BCT, blood collection tube; bp, base pairs; cfDNA, circulating free DNA; ccfDNA, circulating, cell-free DNA; ctDNA, circulating tumor DNA; ddPCR, digital drop pPCR; EDTA, ethylenediaminetetraacetic acid; IASLC, International Association for the Study of Lung Cancer; LB, liquid biopsy; NSCLC, non-small-cell lung cancer; TB, tissue biopsy.

Recommendations for the analytical validation of NGS-based ctDNA assays were recently published by Godsey and colleagues.^13^ These include the assessment of multiple parameters such as technical sensitivity and specificity.^13^ Typically, these are measured using reference materials containing variants of known frequency.^13^ Technical sensitivity can be defined as the upper limit of detection of an assay, where the technical specificity relates to the ability for an assay to offer accurate molecular profiling for a single mutation or panel of genes.^37^^,^^38^ Clinical sensitivity is used to indicate the capability of the test to identify patients who respond to a treatment, or to predict outcome correctly.^37^^,^^38^ Meanwhile, clinical specificity is frequently used to indicate the accuracy of the test at the associated sensitivity.^37^^,^^38^ In ctDNA mutation analysis, a higher clinical sensitivity can be obtained by dropping the VAF threshold, but this comes at the expense of reduced specificity as the number of false positives increases.^37^^,^^38^ A way to represent clinical assay performance is therefore to use positive and negative predictive values (PPVs and NPVs, respectively) at a set limit of detection. At a given technical threshold, an assay will have a clinical PPV (the percentage of patients predicted to respond/have a particular outcome and who actually did) and NPV (the percentage of patients who were predicted not to respond and who did not). High PPV equates to low false negatives, and a high NPV means low false positives.^37^^,^^38^

Recent data from various intra- and inter-laboratory comparisons indicate a substantial variability of NGS assays with respect to analytical sensitivity and specificity, highlighting the importance of extensive validation of the test performance before offering these tests in clinical practice.^22^^,^^25^^,^^39^^,^^40^ In interlaboratory comparisons using vendor assays, mutations were easily identified down to 1% allele frequency, whereas detection at 0.1% proved challenging.^25^^,^^39^^,^^40^ Half of true positives, and most false negatives and false-positive calls, were found below 1% VAF, indicating the need for improved assay performance below this threshold.^25^^,^^39^^,^^40^ Likewise, a recent multisite, cross-platform evaluation of the analytical performance of five industry-leading ctDNA assays demonstrated a high sensitivity, precision, and reproducibility for VAF above 0.5%.^22^ However, for variants below this limit, detection became unreliable, especially when input material was limited.^22^ Discordance was mainly due to technical variations and biological factors such as clonal hematopoiesis of indeterminate potential (CHIP) and tumor heterogeneity.^39^ Moreover, sampling error can occur when counting the number of fragments in the assay at a certain level, meaning that a mutation may be present in one analysis and not in another.^27^ These limits have recently been supported by the SEQC2 international sequencing quality consortium evaluating the technical performance of ctDNA assays.^22^

All these inter- and intra-assay comparisons stress the importance of external quality assessment (EQA) participation, a critical aspect of laboratory quality management, because EQA participation is mandatory to be accredited.^41^ By now several EQA programs exist for ctDNA mutation testing, including Gen&Tiss, GenQA, and ESP/IQNPath; however, despite the existence of these programs, it is challenging to achieve consensus on what to test, the technique needed, and how this differs per individual genetic target.

A pilot project demonstrated the feasibility of establishing a ctDNA mutation testing EQA scheme and highlighted the need for such actions in light of high error rates in detecting clinically relevant low-frequency variants.^22^^,^^42^ Industry, diagnostic laboratories, and quality assurance organizations need to work together to standardize and validate ways of testing. There should be consensus on a minimal set of parameters to report and how to report them, to ensure comparability of results across centers. As not all laboratories work in the same ways, and to ensure innovation, standardization may be premature at present, based on the ever-changing clinical and technological landscape, but it is an important consideration for the future. The Netherlands COIN consortium addresses issues associated with pre- and post-analytical steps, healthcare technology assessment, decision support, and reimbursement issues in a multidisciplinary fashion.^43^ This team works to develop stepwise, evidence-based implementation of ctDNA in routine clinical care, a best-practice example that may be applicable for other laboratories internationally.^43^

The field is building on tests that target single genes to whole-genome sequencing-based panels and, more recently, combined mutational, methylation, and proteomic profiling approaches.^28^^,^^44^ It is important to understand what will be needed in terms of clinical application, given that gene-panel and whole-genome sequencing approaches are, for the moment, considerably more costly and complex than their single-gene or hotspot counterparts.^28^ Complex and simple approaches can be applied separately or together to provide the necessary information, dependent on the patient need. As the choice of method is also very much dependent on the specific application, consensus is needed on which methodologies may provide the best answers at various stages of the patient journey.^4^^,^^21^

## Somatic mosaicism in plasma can be a challenge

Clonal hematopoiesis (CH) involves the accumulation of somatic mutations in hematopoietic stem cells. This leads to clonal expansion of mutations in blood cells and may account for non-tumor-derived mutations detected in the plasma. CH is associated with the normal process of aging in both healthy individuals and those with cancer.^45^, ^46^, ^47^ CHIP usually refers to mutations in driver genes.^45^, ^46^, ^47^ There is increasing evidence of CH mutations in genes usually mutated in solid tumors, including *TP53*, *KRAS*, *GNAS*, *NRAS*, and *PIK3CA.*^45^, ^46^, ^47^ CHIP variants in hematological malignancies are classified as mutations with a minimum VAF of 2%.^45^, ^46^, ^47^ However, in solid malignancies, VAFs of CH-related variants can be much lower (between 0.1% and 0.5%), and often overlap with the range of ctDNA-derived variants.^45^ Therefore these variants represent important natural biological confounders. Recent studies of patients with NSCLC reported presence of CH-related variants in >50% of patients, with up to 20% of detected variants being CH derived and without a matched peripheral blood mononuclear cell (PBMC) analysis; these variants would have been mistakenly identified as tumor-derived variants.^31^^,^^32^^,^^48^^,^^49^

Whether or not PBMCs should be sequenced to correct for CH-related variants is still a matter of debate, mainly due to the extra costs involved in paired sequencing. For focused panels interrogating well-defined, clinically relevant mutations, it might not be necessary, because hotspot oncogenic events that are relevant for solid malignancies are usually not causative for CH.^50^^,^^51^ However, at a panel scale, incorrect classification of CH mutations as tumor-derived mutations could lead to inappropriate therapeutic management.^45^ One benefit of including PBMCs might be a concurrent assessment of precursors of (therapy-related) hematological malignancies, given that clear cut-offs will be established for determining the relative or absolute risk of developing such diseases.^45^^,^^52^

In addition to a paired plasma and PBMC analysis, variant filters based on association of a mutation with CHIP as well as functional annotation of a somatic variant as an oncogene activating event can be used to filter for CH-related variants.^53^ A better understanding of biophysical features (e.g. fragment size and fragmentation patterns) of CH-related variants with the integration of machine learning may reduce the need to perform white blood cell-paired sequencing.^31^^,^^45^

## Variant interpretation and reporting

An important step in the delivery of precision oncology to patients with lung cancer is the interpretation and reporting of variants in the clinical context.^54^ However, there are various levels of complicating factors, starting with distinguishing between variants that might not be disease-related, for example, CH-related variants, PCR/sequencing artifacts, and passenger mutations, and proceeding to interpreting low VAF variants or putative predisposing germline variants, as well as defining actionable mutations and the associated therapy decision.^54^

### Low VAF

With NSCLC being a low ctDNA tumor entity,^55^ interpreting low VAF variants can be the most challenging aspect of reliably reporting ctDNA results.^45^^,^^56^ In particular, in patients with low disease burden or with bone or brain metastasis, circulating free DNA (cfDNA) quantities may be low. Tumor heterogeneity associated with subclonal variants aggravates the situation. Moreover, some specific mutations can be under-representative of their frequency in tumors such as *KRAS* G12.^57^ It is unknown whether variants at low allele fractions are as responsive to targeted therapy as those at high allele fractions. Some studies indicated that low VAF oncogenic drivers respond to targeted therapy, which serves to emphasize the need for highly sensitive tests when treating patients with NSCLC.^58^^,^^59^ Furthermore, these data suggest that targeted treatment response for driver mutations detected by cfDNA may be independent of VAF.^58^ However, evidence also suggests several advantages of reporting increased VAF over time, given that such an increase could reflect poor prognosis.^11^ VAF kinetics in longitudinal studies may be more important in addition to the quantification of cfDNA, and repeat observation will increase confidence in testing.^50^ In addition, reporting on ctDNA mutation levels per milliliter of plasma as well as VAF reporting may be beneficial in case of confounding factors, such as incorrect processing or technical errors.^25^^,^^27^^,^^60^

### Incidental germline cancer predisposition mutations

Although lung cancer is not commonly associated with hereditary cancer susceptibility and is environmentally provoked, a subset of lung adenocarcinoma patients (2.5%-4.5%) may be linked to germline variants in well-known predisposing cancer genes such as *ATM* (50%), *BRCA2,* or *TP53*.^61^ Besides, germline *EGFR* mutations (e.g. T790M) were reported that may be difficult to discriminate from their targetable somatic counterpart in ctDNA mutation testing when no PBMC analysis is included in parallel.^62^ Like with tumor testing, with an increasing number of genetic tests as well as the analyzed gene, the likelihood of detecting germline variants also increases.^63^ Therefore, test providers and physicians need to inform patients that, although the majority of detected alterations represent somatic events, ctDNA mutation testing may identify incidental germline mutations. Currently there is no consensus on how to deal with such findings. Molecular reports should at least include information about the need for confirmatory testing, genetic counseling services, and/or education materials.

### Identification of actionable targets and therapy matching

Another key challenge includes which specific alterations should be classified as ‘actionable’. To support classification, the Association for Molecular Pathology (AMP), American Society of Clinical Oncology (ASCO), and College of American Pathologists (CAP) jointly published a four-tiered system classification system for the interpretation and reporting of sequence variants in cancer.^26^ The European Society for Medical Oncology (ESMO) also recommends the ESMO Scale for Clinical Actionability of Molecular Targets (ESCAT) variant classification guidelines, with subtle differences from the AMP/ASCO/CAP Guidelines.^64^

Moreover, for clinical decision making, a variety of tools and platforms have recently been developed. Ever-growing knowledge bases such as the Cancer Genome Interpreter Cancer Biomarkers Database (CGI), Clinical Interpretation of Variants in Cancer (CIViC), Jackson Laboratory Clinical Knowledgebase (JAX-CKB), MolecularMatch (MMatch), OncoKB, and the Precision Medicine Knowledgebase (PMKB) facilitate interpretation. To overcome the dramatic differences in the components of variant interpretations, efforts are being made to develop a framework for structuring and harmonizing clinical interpretations across these knowledge bases.^65^ Moreover, several commercial decision support platforms exist to help physicians define targetability, but comparisons between such platforms demonstrated a high variability of treatment recommendation.^21^

The final call for choice of therapy is still made by a molecular tumor board, and decision support software tools can support the tumor board’s discussion and ultimately clinician’s decision on treatment strategy.^21^ In addition, an interdisciplinary contribution from a molecular tumor board was suggested to help train clinicians and explain what needs to be considered when utilizing reports. Sharing information on national level could be beneficial for difficult cases and could help to standardize ways of working. Research into harmonizing interpretation of variant evaluation highlights that there is a key need for open, interoperable sharing of variant interpretation data to improve consensus and allow for efficient decision making on sequencing analysis in oncology.^65^^,^^66^

### Reporting of molecular profiling results

Certain minimum requirements are needed for the reporting of molecular profiling results for all CAP-accredited laboratories.^11^ In Europe, this level of guidance is also recommended by IQNPath—representing most EQA providers following recommendations of ISO 15189.^55^ These requirements cover assay methodology, basic clinical performance characteristics including clinical and analytical sensitivity and specificity, assay results, and interpretation [covering the test used and Human Genome Variation Society (HGVS) annotations]. However, additional parameters should be incorporated into the molecular report, such as including primary driver mutations and aberrations present, recent clinical studies/potential off-label treatment, a second set of likely mutations and potential targets that are not fully validated, if there are no clinically relevant mutations present in the patient, and—most importantly—a clinical interpretation.^67^ Optional extra information can include a detailed account of the technical sensitivity and validation protocols that have been used. For ctDNA mutation testing, an overall estimate of the tumor fractions (if available, e.g. from larger gene panels with many mutations detected) could be helpful for the interpretation of individual variants. The allelic fraction, absolute quantification of mutation (in copies/ml plasma), assay uncertainty and methodology, and minimal quality control metrics (coverage, read depth, etc.) of the run/sample are also suggested.

## Liquid profiling in patients with metastatic NSCLC

**Figure 3:**
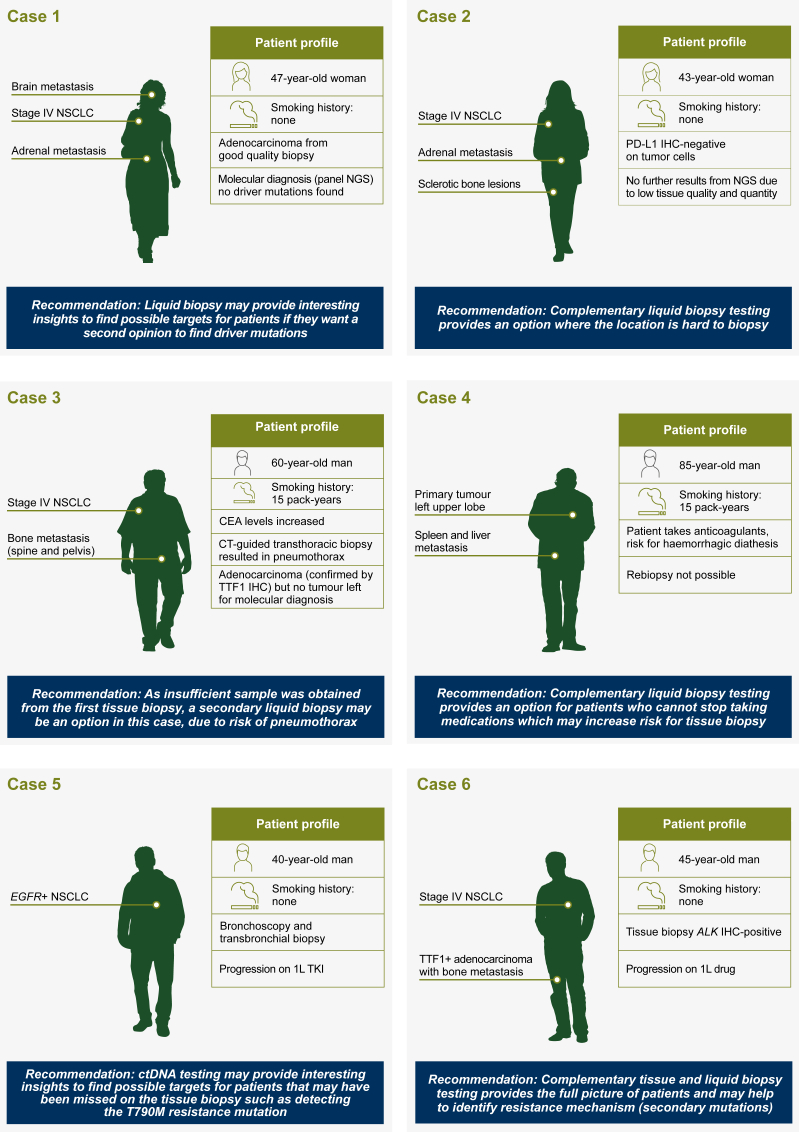
**Hypothetical patient scenarios for the use of ctDNA mutation testing in metastatic NSCLC.** 1L, first line; ALK, anaplastic lymphoma kinase; BRAF, proto-oncogene B-Raf; CEA, carcinoembryonic antigen; CT, computed tomography; ctDNA, circulating tumor DNA; EGFR, epidermal growth factor receptor; IHC, immunohistochemistry; MET, proto-oncogene tyrosine-protein kinase met; NGS, next-generation sequencing; NSCLC, non-small cell lung cancer; NTRK, neurotrophic tyrosine receptor kinase; PD-L1, programmed death-ligand 1; RET, ret proto-oncogene; ROS1, proto-oncogene tyrosine-protein kinase ROS; TKI, tyrosine kinase inhibitor; TTF1, transcription termination factor 1.

Approximately one-third of patients with advanced NSCLC may die within the first 2 months after initial diagnosis.^68^ The poor Eastern Cooperative Oncology Group performance status of many patients with advanced NSCLC may also limit the role of uncomfortable, on occasion dangerous, interventional biopsy procedures, as well as the need for significantly shorter turnaround times, which plasma testing can offer both at primary diagnosis and at progression.^8^^,^^69^ Up to 80% of patients with NSCLC having advanced disease will only have tissue from small biopsies or cytology samples, limiting the potential to perform additional tests.^2^^,^^8^^,^^70^ Reports show that up to 31% of patients do not have accessible tissue, and up to 20% of biopsies are inadequate for molecular testing due to insufficient tissue amounts, which highlights the need for easily accessible, minimally invasive complementary testing techniques.^2^^,^^8^^,^^70^ Sequencing of ctDNA can efficiently identify genomic targets in advanced NSCLC, and clinical outcomes in patients who have been treated with targeted therapy based on actionable alterations detected by ctDNA are consistent with those reported based on tissue profiling.^71^^,^^72^ In Figure 3 we present specific hypothetical use cases to provide a suggested practical framework for the implementation of LB (specifically ctDNA mutation testing) in clinical practice. When referring to the suggested cases, please ensure adherence to any relevant institute- and country-specific guidelines.

In cases with discordant clinical history (e.g. nonsmoker but squamous cell carcinoma) of suspected inter- and intra-tumor heterogeneity, cfDNA testing may be a good option. The usefulness of LB in oncogene-addicted first-line TKI refractoriness is linked to availability (in clinical practice or trials) of a specific drug to overcome the resistance.^18^^,^^73^ In one-third of TKI-resistant cases with plasma testing alone, a therapeutically targetable mutation was detected.^19^ Therefore, in cases without a druggable target from tissue, ctDNA might provide additional therapeutic options if patients require a second opinion (Figure 3, case 1).

Moreover, ctDNA analysis may have additional benefit in patients who have inappropriate locations for samples to be taken, such as lesions within the bone or adrenal glands (Figure 3, case 2) or if there is a risk of complications (Figure 3, case 3). The rate of complication from pneumothorax after lung biopsy was calculated to be in the range of 17%-38%.^74^, ^75^, ^76^ Other complications exist, even some mortalities in case of rebiopsy; therefore using LB could dramatically reduce such complications (Figure 3, cases 3 and 4).

On occasion, ctDNA mutation testing can reveal mutations that are not present in the initial TB—one case reveals discordant genotypes between tumor biopsy and blood-based analyses that may result from technological differences, as well as sampling different tumor cell populations (Figure 3, case 5).^73^ Acquisition of adequate tissue in patients who relapse after first-line targeted treatment can be challenging; complementary ctDNA mutation testing could help to provide useful information about the tumor by detecting molecular mechanisms of resistance (Figure 3, case 6).

Testing treatment-naive patients for possible targets is also of interest, in particular if time matters or if TB is refused. A patient questionnaire revealed that up to 33% of participants rejected a rebiopsy, mainly due to initial biopsy being painful. Furthermore, up to 40% of the cohort would consider a noninvasive option if it were made available to them.^77^ It is of note that ctDNA analysis from blood is challenging in patients with brain metastasis, which still affects 24%-44% of patients. Because the blood–brain barrier prevents ctDNA from entering the blood, an alternative source of ctDNA can also be found in the cerebrospinal fluid, which is of particular importance when evaluating the molecular characteristics of brain metastases. Sequencing of ctDNA found in the cerebrospinal fluid showed specific mutation patterns in driver genes among people who had NSCLC and brain metastasis.^78^

Taken together, there is strong evidence to support the inclusion of LB in the management of NSCLC during the early treatment phase.^79^ ctDNA analysis is considered to be reliable and adequate to initiate first-line targeted therapy for detection of mutated genes and rearrangements.^11^^,^^17^ Steps are needed to translate this into clinical practice, including proof-of-concept studies.^80^ There is not a ‘one-size-fits-all’ ctDNA approach; it will rather be necessary to consider patients on a case-by-case basis. Although a plasma-first approach seems tempting and, in many cases, would yield faster diagnosis and shorter time to targeted treatment, sometimes only a combination of both testing modalities (tissue and plasma) will provide a complete picture.^11^

## Other potential clinical applications in patients with NSCLC

ctDNA can have a role to play for treatment decision making, but also other potential applications are emerging (Supplementary Table S2, available at https://doi.org/10.1016/j.esmoop.2022.100399).^79^ As a high level of ctDNA is typically linked to a worse survival, ctDNA can be used to estimate prognosis.^9^^,^^10^ A reduction in ctDNA mutation frequencies during treatment is predictive of response and can be leveraged for monitoring purposes.^9^^,^^10^ Furthermore, the predictive value of ctDNA for assessing and monitoring of response during immune checkpoint inhibitor treatment is currently being intensively investigated.^1^^,^^80^ ctDNA levels have also recently been linked to response to immunotherapy in breast cancer.^81^

Moreover, ctDNA is an adjuvant biomarker capable of both detecting MRD following surgery and defining the clonality of relapsing disease. The persistence of ctDNA after surgical resection of a primary tumor may be indicative of disease recurrence.^13^^,^^82^, ^83^, ^84^ In this context, the impact of taking the blood at specific time points after surgery is a key consideration for MRD and can assist when monitoring treatment response.^85^ Increasing workflows toward MRD testing could stimulate the development of assays in a tumor-guided sequencing approach to improve earlier detection of cancer.^86^^,^^87^ These data pave the way for clinical trials predicated on escalation of adjuvant standard of care in patients with cancer who exhibit MRD-positive status following surgery.^82^^,^^84^ Yet, further evidence generation is needed to support the use of LB in MRD testing, including data showing that patients who undergo LB testing perform as well as or better than those without LB testing.^4^^,^^84^

## Current ctDNA mutation testing landscape and reimbursement

In some countries, reimbursement is in place for ctDNA mutation testing (i.e. *EGFR* testing); however, reimbursement for broad coverage is limited.^30^ The authors are aware that this is reflective of their areas of practice and acknowledge there may be differences in local areas. In order to improve reimbursement profiles, there are many points to consider, including the following: more interventional data are needed as often data are retrospective; studies should detail clinical cut-offs for VAF; further guidance regarding flexible, standardized workflows is needed, which may facilitate uptake in laboratories and improve the quality of testing (with overall guidance for laboratories to follow, but not limited to certain tests); and more health economics data are needed to highlight the cost-effectiveness of LB testing.^88^^,^^89^

These data and others suggest that inclusion of plasma testing is imperative to increase access to personalized care and improve cost-effectiveness for patients with NSCLC.^89^ Bypassing histopathology can have many advantages for cost, time, and getting patients onto treatment more rapidly. However, if histopathological procedures are supplemented or replicated rather than replaced, many of these arguments are obviated. A proper study of best practice in sample use for diagnostics is required. Overall, LB testing displays relatively low costs compared with treatment costs, and may improve patient diagnosis and disease management.^88^

## Outlook

The most important aspect of blood testing is the possibility to detect and diagnose cancerous lesions sooner and even at earlier stages of the disease. As studies into this mature, they may transform how we approach and treat cancer as a disease. Such studies involving research into multianalyte blood tests for various cancer types are conceptually and practically being explored with promising results regarding ultrasensitive testing (e.g. CancerSEEK) to enhance early detection by LB screening.^90^ Several new approaches are under development, based on the analysis of methylation, cfDNA fragmentation, and proteomic profiling, or combinations of multiple analytes to increase the clinical sensitivity.^91^, ^92^, ^93^, ^94^ In terms of metastatic NSCLC, this type of test suggests that it is possible to detect solid tumors at an earlier stage with a combination of ctDNA mutation testing and protein biomarkers to help early detection of those at risk of developing metastases.^90^ Accounting for cfDNA biology could improve the design and performance of LB assays. cfDNA fragments from a variety of cells and tissues tend to be shorter than cfDNA derived from hematopoietic cells by ∼25 bp.^91^^,^^95^ The biological shift of size of cfDNA can be leveraged using machine learning classifiers to improve the detection of multiple cancer types.^96^^,^^97^

ctDNA mutant allele fractions can be extremely low, so mutation-based detection of ctDNA has clear limitations for MRD and monitoring within the plasma.^91^^,^^98^ New approaches are under development to further increase the sensitivity of ctDNA testing.^91^, ^92^, ^93^^,^^98^ Explorations into MRD, early detection of high-risk individuals, and potential trial enrollment following therapy exhaustion are ongoing.^29^^,^^99^ Key trials supporting LB testing in this segment of the patient journey are also included in Supplementary Table S2, available at https://doi.org/10.1016/j.esmoop.2022.100399.

It should be mentioned that other LB analytes, such as circulating tumor cells, circulating microRNAs, tumor-educated platelets, or tumor-associated proteins, might provide complementary information for patients with cancer.^60^

The future of ctDNA LB testing opens up many potential avenues to assist in both research- and clinical-based settings. Currently the field utilizes ctDNA to identify single genes and is evolving to integrate omics data from proteomes or genome-wide sequencing where needed—combined application tests are not currently needed to address clinically relevant applications.^94^ Integrating data from multiple tumor-associated materials can be further enhanced by combination with lifestyle or medical imaging data with the help of artificial intelligence, ultimately to improve efficiency and detection rates of certain cancer types.^94^

## Conclusions

Proper implementation of ctDNA mutation testing in routine care can be envisioned in the near future. As the clinical evidence of utility expands, the use of LB alongside tissue sample analysis may occur in the patient cases detailed above.

ctDNA LB mutation testing offers both challenges and benefits.^5^^,^^13^ Where TB is not possible, the recommendations for use in clinical practice can provide insight into how ctDNA LB may provide clinical utility. However, today ctDNA LB can also provide quick, easy, complementary testing modalities for patients with metastatic NSCLC, and may be selected in specific clinical scenarios.

As the clinical evidence of utility expands, routine molecular profiling using ctDNA LB alongside tissue sample analysis improves clinical sensitivity in clinical practice for improved treatment decision making for people with NSCLC.

## Supplementary data

Supplementary Tables S1 and s2 and Supplementary References

## References

[1] Guibert N., Pradines A., Favre G. (2020). Current and future applications of liquid biopsy in nonsmall cell lung cancer from early to advanced stages. Eur Respir Rev.

[2] Liam C.K., Mallawathantri S., Fong K.M. (2020). Is tissue still the issue in detecting molecular alterations in lung cancer?. Respirology.

[3] Scherer F. (2020). Capturing tumor heterogeneity and clonal evolution by circulating tumor DNA profiling. Recent Results Cancer Res.

[4] Wan J.C.M., Massie C., Garcia-Corbacho J. (2017). Liquid biopsies come of age: towards implementation of circulating tumour DNA. Nat Rev Cancer.

[5] Raja R., Kuziora M., Brohawn P.Z. (2018). Early reduction in ctDNA predicts survival in patients with lung and bladder cancer treated with durvalumab. Clin Cancer Res.

[6] National Comprehensive Cancer Network (NCCN) (2021). NCCN Clinical Practice Guidelines in Oncology (NCCN Guidelines®): Non-small cell lung cancer. Version: 7.2021. https://www.nccn.org/professionals/physician_gls/PDF/nscl.pdf.

[7] AstraZeneca A.B. (2020). TAGRISSO (osimertinib). Summary of product characteristics. https://www.ema.europa.eu/en/documents/product-information/tagrisso-epar-product-information_en.pdf.

[8] Ilié M., Hofman P. (2016). Pros: can tissue biopsy be replaced by liquid biopsy?. Transl Lung Cancer Res.

[9] Goldberg S.B., Narayan A., Kole A.J. (2018). Early assessment of lung cancer immunotherapy response via circulating tumor DNA. Clin Cancer Res.

[10] Giroux Leprieur E., Herbretau G., Dumenil C. (2018). Circulating tumor DNA evaluated by next-generation sequencing is predictive of tumor response and prolonged clinical benefit with nivolumab in advanced non-small cell lung cancer. Oncoimmunology.

[11] Rolfo C., Mack P.C., Scagliotti G.V. (2018). Liquid biopsy for advanced non-small cell lung cancer (NSCLC): a statement paper from the IASLC. J Thorac Oncol.

[12] Papadopoulou E., Tsoulos N., Tsantikidi K. (2019). Clinical feasibility of NGS liquid biopsy analysis in NSCLC patients. PLoS One.

[13] Godsey J.H., Silvestro A., Barrett J.C. (2020). Generic protocols for the analytical validation of next-generation sequencing-based ctDNA assays: a joint consensus recommendation of the BloodPAC's Analytical Variables Working Group. Clin Chem.

[14] Paik P.K., Felip E., Veillon R. (2020). Tepotinib in non-small-cell lung cancer with MET exon 14 skipping mutations. N Engl J Med.

[15] Gadgeel S.M., Mok T.S.K., Peters S. (2019). Phase II/III blood-first assay screening trial (BFAST) in treatment-naïve NSCLC: initial results from the ALK+ cohort. Ann Oncol.

[16] Remon J., Lacroix L., Jovelet C. (2019). Real-world utility of an amplicon-based next-generation sequencing liquid biopsy for broad molecular profiling in patients with advanced non-small-cell lung cancer. JCO Precis Oncol.

[17] Aggarwal C., Thompson J.C., Black T.A. (2019). Clinical implications of plasma-based genotyping with the delivery of personalized therapy in metastatic non-small cell lung cancer. JAMA Oncol.

[18] Horn L., Whisenant J.G., Wakelee H. (2019). Monitoring therapeutic response and resistance: analysis of circulating tumor DNA in patients with ALK+ lung cancer. J Thorac Oncol.

[19] Thompson J.C., Yee S.S., Troxel A.B. (2016). Detection of therapeutically targetable driver and resistance mutations in lung cancer patients by next-generation sequencing of cell-free circulating tumor DNA. Clin Cancer Res.

[20] Jiang J., Adams H.P., Yao L. (2020). Concordance of genomic alterations by next-generation sequencing in tumor tissue versus cell-free DNA in stage I-IV non-small cell lung cancer. J Mol Diagn.

[21] Perakis S.O., Weber S., Zhou Q. (2020). Comparison of three commercial decision support platforms for matching of next-generation sequencing results with therapies in patients with cancer. ESMO Open.

[22] Deveson I.W., Gong B., Lai K. (2021). Evaluating the analytical validity of circulating tumor DNA sequencing assays for precision oncology. Nat Biotechnol.

[23] Elazezy M., Joosse S.A. (2018). Techniques of using circulating tumor DNA as a liquid biopsy component in cancer management. Comput Struct Biotechnol J.

[24] Kerr K.M., Bibeau F., Thunnissen E. (2021). The evolving landscape of biomarker testing for non-small cell lung cancer in Europe. Lung Cancer.

[25] Weber S., Spiegl B., Perakis S.O. (2020). Technical evaluation of commercial mutation analysis platforms and reference materials for liquid biopsy profiling. Cancers.

[26] Lindeman N.I., Cagle P.T., Aisner D.L. (2018). Updated molecular testing guideline for the selection of lung cancer patients for treatment with targeted tyrosine kinase inhibitors: guideline from the College of American Pathologists, the International Association for the Study of Lung Cancer, and the Association for Molecular Pathology. Arch Pathol Lab Med.

[27] Strom S.P. (2016). Current practices and guidelines for clinical next-generation sequencing oncology testing. Cancer Biol Med.

[28] Ignatiadis M., Sledge G.W., Jeffrey S.S. (2021). Liquid biopsy enters the clinic - implementation issues and future challenges. Nat Rev Clin Oncol.

[29] Murtaza M., Dawson S.J., Pogrebniak K. (2015). Multifocal clonal evolution characterized using circulating tumour DNA in a case of metastatic breast cancer. Nat Commun.

[30] IJzerman M., de Boer J., Azad A. (2021). Towards routine implementation of liquid biopsies in cancer management: it is always too early, until suddenly it is too late. Diagnostics (Basel).

[31] Chabon J.J., Hamilton E.G., Kurtz D.M. (2020). Integrating genomic features for non-invasive early lung cancer detection. Nature.

[32] Razavi P., Li B.T., Brown D.N. (2019). High-intensity sequencing reveals the sources of plasma circulating cell-free DNA variants. Nat. Med.

[33] Yaung S.J., Fuhlbrück F., Peterson M. (2020). Clonal hematopoiesis in late-stage non–small-cell lung cancer and its impact on targeted panel next-generation sequencing. JCO Precis Oncol.

[34] Sorber L., Zwaenepoel K., De Winne K. (2018). A multicenter study to assess *EGFR* mutational status in plasma: focus on an optimized workflow for liquid biopsy in a clinical setting. Cancers.

[35] European Medicines Agency (EMA), Committee for Medicinal Products for Human Use (CHMP) (2018). Guideline on good pharmacogenomic practice. EMA/CHMP/718998/2016. https://www.ema.europa.eu/en/documents/scientific-guideline/guideline-good-pharmacogenomic-practice-first-version_en.pdf.

[36] International Organization for Standardization (ISO) (2017). International Electrotechnical Commission (IEC). ISO/IEC 17025: general requirements for the competence of testing and calibration laboratories. https://www.iso.org/files/live/sites/isoorg/files/store/en/PUB100424.pdf.

[37] Poole J.C., Wu S.F., Lu T.T. (2019). Analytical validation of the Target Selector ctDNA platform featuring single copy detection sensitivity for clinically actionable EGFR, BRAF, and KRAS mutations. PLoS One.

[38] Trevethan R. (2017). Sensitivity, specificity, and predictive values: foundations, pliabilities, and pitfalls in research and practice. Front Public Health.

[39] Stetson D., Ahmed A., Xu X. (2019). Orthogonal comparison of four plasma NGS tests with tumor suggests technical factors are a major source of assay discordance. JCO Precis Oncol.

[40] Koessler T., Paradiso V., Piscuoglio S. (2020). Reliability of liquid biopsy analysis: an inter-laboratory comparison of circulating tumor DNA extraction and sequencing with different platforms. Lab Invest.

[41] World Health Organization (WHO), U.S. Centers for Disease Control and Prevention (CDC), Clinical and Laboratory Standards Institute® (2009). Laboratory Quality Management System Training Toolkit. Content Sheet 10-1: Overview of external quality assessment (EQA). https://www.who.int/ihr/training/laboratory_quality/10_b_eqa_contents.pdf.

[42] Keppens C., Dequeker E.M.C., Patton S.J. (2018). International pilot external quality assessment scheme for analysis and reporting of circulating tumour DNA. BMC Cancer.

[43] ZonMw (2020). ctDNA on the way to implementation in the Netherlands (COIN). https://www.zonmw.nl/nl/onderzoek-resultaten/doelmatigheidsonderzoek/programmas/project-detail/goed-gebruik-geneesmiddelen/ctdna-on-the-way-to-implementation-in-the-netherlands-coin/.

[44] Bailleux C., Lacroix L., Barranger E. (2020). Using methylation signatures on cell-free DNA for early cancer detection: a new era in liquid biopsy?. Ann Oncol.

[45] Chan H.T., Chin Y.M., Nakamura Y. (2020). Clonal hematopoiesis in liquid biopsy: from biological noise to valuable clinical implications. Cancers.

[46] Heuser M., Thol F., Ganser A. (2016). Clonal hematopoiesis of indeterminate potential. Dtsch Arztebl Int.

[47] Steensma D.P., Bejar R., Jaiswal S. (2015). Clonal hematopoiesis of indeterminate potential and its distinction from myelodysplastic syndromes. Blood.

[48] Zhang Y., Yao Y., Xu Y. (2021). Pan-cancer circulating tumor DNA detection in over 10,000 Chinese patients. Nat Commun.

[49] Weber S., Leest Pvd, Donker H.C. (2021). Dynamic changes of circulating tumor DNA predict clinical outcome in patients with advanced non–small-cell lung cancer treated with immune checkpoint inhibitors. JCO Precis Oncol.

[50] Liu J., Chen X., Wang J. (2019). Biological background of the genomic variations of cf-DNA in healthy individuals. Ann Oncol.

[51] Hu Y., Ulrich B.C., Supplee J. (2018). False-positive plasma genotyping due to clonal hematopoiesis. Clin Cancer Res.

[52] Wang H., Yin Y., Wang R. (2020). Clinicopathological features, risk and survival in lung cancer survivors with therapy-related acute myeloid leukaemia. BMC Cancer.

[53] Abbosh C., Swanton C., Birkbak N.J. (2019). Clonal haematopoiesis: a source of biological noise in cell-free DNA analyses. Ann Oncol.

[54] Malone E.R., Oliva M., Sabatini P.J.B. (2020). Molecular profiling for precision cancer therapies. Genome Med.

[55] Deans Z.C., Butler R., Cheetham M. (2019). IQN path ASBL report from the first European cfDNA consensus meeting: expert opinion on the minimal requirements for clinical ctDNA testing. Virchows Archiv.

[56] Mizuno K., Akamatsu S., Sumiyoshi T. (2019). eVIDENCE: a practical variant filtering for low-frequency variants detection in cell-free DNA. Sci Rep.

[57] Chen I., Raymond V.M., Geis J.A. (2017). Ultrasensitive plasma ctDNA KRAS assay for detection, prognosis, and assessment of therapeutic response in patients with unresectable pancreatic ductal adenocarcinoma. Oncotarget.

[58] Jacobs M.T., Mohindra N.A., Shantzer L. (2018). Use of low-frequency driver mutations detected by cell-free circulating tumor DNA to guide targeted therapy in non–small-cell lung cancer: a multicenter case series. JCO Precis Oncol.

[59] Helman E., Nguyen M., Karlovich C.A. (2018). Cell-free DNA next-generation sequencing prediction of response and resistance to third-generation EGFR inhibitor. Clin Lung Cancer.

[60] Keller L., Belloum Y., Wikman H. (2021). Clinical relevance of blood-based ctDNA analysis: mutation detection and beyond. Br J Cancer.

[61] Parry E.M., Gable D.L., Stanley S.E. (2017). Germline mutations in DNA repair genes in lung adenocarcinoma. J Thorac Oncol.

[62] Yamamoto H., Yatabe Y., Toyooka S. (2018). Inherited lung cancer syndromes targeting never smokers. Transl Lung Cancer Res.

[63] Slavin T.P., Banks K.C., Chudova D. (2018). Identification of incidental germline mutations in patients with advanced solid tumors who underwent cell-free circulating tumor DNA sequencing. J Clin Oncol.

[64] Mateo J., Chakravarty D., Dienstmann R. (2018). A framework to rank genomic alterations as targets for cancer precision medicine: the ESMO Scale for Clinical Actionability of molecular Targets (ESCAT). Ann Oncol.

[65] Wagner A.H., Walsh B., Mayfield G. (2020). A harmonized meta-knowledgebase of clinical interpretations of somatic genomic variants in cancer. Nat Genet.

[66] Leichsenring J., Horak P., Kreutzfeldt S. (2019). Variant classification in precision oncology. Int J Cancer.

[67] Mosele F., Remon J., Mateo J. (2020). Recommendations for the use of next-generation sequencing (NGS) for patients with metastatic cancers: a report from the ESMO Precision Medicine Working Group. Ann Oncol.

[68] Globus O., Bar J., Onn A. (2019). Early mortality in metastatic lung cancer: a SEER population data analysis. J Clin Oncol.

[69] Sholl L.M., Aisner D.L., Allen T.C. (2016). Liquid biopsy in lung cancer: a perspective from members of the Pulmonary Pathology Society. Arch Pathol Lab Med.

[70] Chouaid C., Dujon C., Do P. (2014). Feasibility and clinical impact of re-biopsy in advanced non-small-cell lung cancer: a prospective multicenter study in a real-world setting (GFPC study 12-01). Lung Cancer.

[71] Mack P.C., Banks K.C., Espenschied C.R. (2020). Spectrum of driver mutations and clinical impact of circulating tumor DNA analysis in non-small cell lung cancer: analysis of over 8000 cases. Cancer.

[72] Remon J., Swalduz A., Planchard D. (2020). Outcomes in oncogenic-addicted advanced NSCLC patients with actionable mutations identified by liquid biopsy genomic profiling using a tagged amplicon-based NGS assay. PLoS One.

[73] Sundaresan T.K., Sequist L.V., Heymach J.V. (2016). Detection of T790M, the acquired resistance EGFR mutation, by tumor biopsy versus noninvasive blood-based analyses. Clin Cancer Res.

[74] Heerink W.J., de Bock G.H., de Jonge G.J. (2017). Complication rates of CT-guided transthoracic lung biopsy: meta-analysis. Eur Radiol.

[75] Lee S.M., Park C.M., Lee K.H. (2014). C-arm cone-beam CT-guided percutaneous transthoracic needle biopsy of lung nodules: clinical experience in 1108 patients. Radiology.

[76] Takeshita J., Masago K., Kato R. (2015). CT-guided fine-needle aspiration and core needle biopsies of pulmonary lesions: a single-center experience with 750 biopsies in Japan. AJR Am J Roentgenol.

[77] Fukui T., Ishihara M., Kasajima M. (2019). Questionnaire survey on patient awareness of invasive rebiopsy in advanced non-small cell lung cancer. Thorac Cancer.

[78] Ma C., Yang X., Xing W. (2020). Detection of circulating tumor DNA from non-small cell lung cancer brain metastasis in cerebrospinal fluid samples. Thorac Cancer.

[79] Kunnath A.P., Priyashini T. (2019). Potential applications of circulating tumor DNA technology as a cancer diagnostic tool. Cureus.

[80] Boonstra P.A., Wind T.T., van Kruchten M. (2020). Clinical utility of circulating tumor DNA as a response and follow-up marker in cancer therapy. Cancer Metastasis Rev.

[81] Powles T., Assaf Z.J., Davarpanah N. (2021). ctDNA guiding adjuvant immunotherapy in urothelial carcinoma. Nature.

[82] Chin R.I., Chen K., Usmani A. (2019). Detection of solid tumor molecular residual disease (MRD) using circulating tumor DNA (ctDNA). Mol Diagn Ther.

[83] Abbosh C., Frankell A., Garnett A. (2020). Phylogenetic tracking and minimal residual disease detection using ctDNA in early-stage NSCLC: a lung TRACERx study. Cancer Res.

[84] Schraa S.J., van Rooijen K.L., van der Kruijssen D.E.W. (2020). Circulating tumor DNA guided adjuvant chemotherapy in stage II colon cancer (MEDOCC-CrEATE): study protocol for a trial within a cohort study. BMC Cancer.

[85] Symonds E.L., Pedersen S.K., Murray D.H. (2018). Circulating tumour DNA for monitoring colorectal cancer—a prospective cohort study to assess relationship to tissue methylation, cancer characteristics and surgical resection. Clin Epigenetics.

[86] Wan J.C.M., Heider K., Gale D. (2020). ctDNA monitoring using patient-specific sequencing and integration of variant reads. Sci Transl Med.

[87] Abbosh C., Birkbak N.J., Wilson G.A. (2017). Phylogenetic ctDNA analysis depicts early-stage lung cancer evolution. Nature.

[88] Cheng M., Akalestos A., Scudder S. (2020). Budget impact analysis of EGFR mutation liquid biopsy for first- and second-line treatment of metastatic non-small cell lung cancer in Greece. Diagnostics (Basel).

[89] Araujo L.H., Timmers C., Bell E.H. (2015). Genomic characterization of non-small-cell lung cancer in African Americans by targeted massively parallel sequencing. J Clin Oncol.

[90] Cohen J.D., Li L., Wang Y. (2018). Detection and localization of surgically resectable cancers with a multi-analyte blood test. Science.

[91] Van der Pol Y., Mouliere F. (2019). Toward the early detection of cancer by decoding the epigenetic and environmental fingerprints of cell-free DNA. Cancer Cell.

[92] Liu M.C., Oxnard G.R., Klein E.A. (2020). Sensitive and specific multi-cancer detection and localization using methylation signatures in cell-free DNA. Ann Oncol.

[93] Chen X., Gole J., Gore A. (2020). Non-invasive early detection of cancer four years before conventional diagnosis using a blood test. Nat Commun.

[94] Heitzer E., Haque I.S., Roberts C.E.S. (2019). Current and future perspectives of liquid biopsies in genomics-driven oncology. Nat Rev Genet.

[95] Underhill H.R., Kitzman J.O., Hellwig S. (2016). Fragment length of circulating tumor DNA. PLoS Genet.

[96] Mouliere F., Chandrananda D., Piskorz A.M. (2018). Enhanced detection of circulating tumor DNA by fragment size analysis. Sci Transl Med.

[97] Cristiano S., Leal A., Phallen J. (2019). Genome-wide cell-free DNA fragmentation in patients with cancer. Nature.

[98] Abbosh C., Birkbak N.J., Swanton C. (2018). Early stage NSCLC – challenges to implementing ctDNA-based screening and MRD detection. Nat Rev Clin Oncol.

[99] Moding E.J., Liu Y., Nabet B.Y. (2020). Circulating tumor DNA dynamics predict benefit from consolidation immunotherapy in locally advanced non-small-cell lung cancer. Nat Cancer.

[100] (2020). Roche Diagnostics. cobas® EGFR Mutation Test v2 (CE-IVD) now approved for use with Roche’s Cell-Free DNA Collection Tube (CE-IVD). https://diagnostics.roche.com/global/en/news-listing/2020/cobas-egfr-mutation-test-v2-approved-for-use-with-roche-cfdna-collection-tube.html.

[101] Baldacchino S. (2021). Current advances in clinical application of liquid biopsy. In: Histopathology and Liquid Biopsy. Rijeka, Croatia: IntechOpen.

[102] EntroGen Inc EGFR Mutation Analysis Kit for Real-Time PCR. http://entrogen.com/web3/egfr-mutation-analysis-kit/.

[103] Genetic Engineering & Biotechnology News (GEN). AmoyDx Gains CE Mark for EGFR, KRAS, and BRAF Tests. February 25, 2011 [press release]. https://www.genengnews.com/topics/translational-medicine/amoydx-gains-ce-mark-for-egfr-kras-and-braf-tests/.

[104] Foundation Medicine Inc FoundationOne® CDx comparability to FoundationOne® [EU report]. https://www.foundationmedicine.se/content/dam/rfm/sv_SE/Documents/EU_F1CDx_Comparability_Letter_low_res.pdf.

[105] Foundation Medicine Inc FoundationOne® Liquid CDx Technical Information. https://info.foundationmedicine.com/hubfs/FMI%20Labels/FoundationOne_Liquid_CDx_Label_Technical_Info.pdf.

[106] Guardant Health Inc Guardant360 CDX Gene list. https://guardant360cdx.com/genelist/.

[107] Normanno N., Denis M.G., Thress K.S. (2017). Guide to detecting epidermal growth factor receptor (EGFR) mutations in ctDNA of patients with advanced non-small-cell lung cancer. Oncotarget.

[108] Gerber T., Taschner-Mandl S., Saloberger-Sindhöringer L. (2020). Assessment of pre-analytical sample handling conditions for comprehensive liquid biopsy analysis. J Mol Diagn.

[109] Bronkhorst A.J., Ungerer V., Holdenrieder S. (2019). The emerging role of cell-free DNA as a molecular marker for cancer management. Biomol Detect Quantif.

[110] Van der Leest P., Boonstra P.A., Elst A.T. (2020). Comparison of circulating cell-free DNA extraction methods for downstream analysis in cancer patients. Cancers.

[111] Lampignano R., Neumann M.H.D., Weber S. (2020). Multicenter evaluation of circulating cell-free DNA extraction and downstream analyses for the development of standardized (pre)analytical work flows. Clin Chem.

