# Supplementary material for: Recommendations for a practical implementation of circulating tumor DNA mutation testing in metastatic non-small-cell lung cancer
Source: ESMO Open. 2022 Feb 21;7(2):100399. doi: 10.1016/j.esmoop.2022.100399 (PMC8867049; doi:10.1016/j.esmoop.2022.100399)
Supplement: Supplementary Tables S1 and s2 and Supplementary References [file mmc1.docx]

|  | Allele-specific PCR | Digital PCR (including ddPCR) | NGS | Broad panel testing | WES/WGS |
| --- | --- | --- | --- | --- | --- |
| What is measured? | Measures defined single nucleotide polymorphisms in a sample | A refined method of allele specific PCR that can identify, amplify, and quantify pre-defined alterations | NGS can make use of ‘hotspot’ tests to identify a limited range of prespecified mutations across several genes, but does not detect all classes of mutations | Broad testing allows the capture of common and rare mutations to allow detailed information about more complex mutational signatures | Whole-exome/-genome sequencing is a comprehensive method for analyzing entire genomes or exomes (protein coding regions of the genome). |
| Key benefits | - Easy to set up - Short time to result - Low cost | - Highly accurate - Short time to result - Low cost of reagents | - Detects multiple classes of mutations (indels, rearrangements, and copy number alterations) - High sensitivity (if deep sequencing methods are used) | - Highly sensitive - Detection of all classes of alterations, genomic signatures and unspecified alterations by genomic signatures (MSI, TMB) | - The whole genome or exome is sequenced, allowing scrutinizing of many genes - Many features can be analysed (fragmentation, SCNA etc.) |
| Key limitations | - Detection of small number of alterations per sample - Lower sensitivity - Uses a large amount of DNA | - Detection of small number of alterations per sample | - Restricted to pre-selected regions of interest - Higher costs - Longer turn-around time | - Higher costs - Longer turn-around time - Interpretation can be difficult - Can reveal variants which are not clinically actionable | - Higher cost associated with the technology - Interpretation can be difficult - Genetic counsel may be needed if off-target genetic mutations are found - High volume of data storage needed |

**SUPPLEMENTARY INFORMATION**

**Table S1: A comparison of technologies for ctDNA mutation testing^1-6^**

ctDNA, circulating tumor DNA; ddPCR, digital droplet PCR; MSI, microsatellite instability; NGS, next-generation sequencing; PCR, polymerase chain reaction; SCNA, somatic copy number alterations; TMB, tumor mutational burden; WES, whole exome sequencing; WGS, whole genome sequencing.

**Table S2.** **Clinical applications of ctDNA liquid biopsy mutation testing in NSCLC**

| **Reference** | **Study population** | **Primary endpoint** | **Key findings** | **Key takeaway** |
| --- | --- | --- | --- | --- |
| Gadgeel *Ann Oncol* 2019^7^ | 2,219 pts with stage IIIB/IV *ALK*+ NSCLC (*ALK* NGS hybrid-capture test x 324 genes) | Confirmed investigator-assessed ORR | Confirmed ORR was 87.4%. Out of 2,219 pts screened, blood-based testing produced results for 2,188 pts, and 119 of these had *ALK*+ | Using blood-based methods to detect *ALK* fusions in NSCLC results in high ORR, validating clinical utility |
| Leighl *Clin Cancer Res* 2019^8^ | 307 pts with biopsy proven, untreated non-squamous stage IIIB/IV mNSCLC, who were undergoing tissue genotyping, with 282 included in analysis (NGS hybrid-capture test x 74 genes) | cfDNA non-inferiority compared to SoC tumor tissue-based genotyping | Genotyping with a well-validated and sensitive cfDNA test can detect biomarkers at a similar rate to SoC tissue genotyping | cfDNA testing results are just as accurate as SoC tissue genotyping and results have a faster turnaround time |
| Schrock *J Thorac Oncol* 2018^9^ | 1,552 pts with advanced NSCLC (NGS hybrid-capture sequencing tests x 62 genes) | The utility of ctDNA mutation testing as a complementary approach tested in advanced NSCLC | Frequency of common variants detected by ctDNA mutation testing was similar to that seen in 21,500 NSCLC tissue samples | Most mutations that were detected in tissue were also detected in ctDNA, demonstrating clinical utility of blood-based testing |
| Abbosh *Nature* 2017^10^ | First 100 pts with early stage lung cancer (Multiplex-PCR NGS test) | To partake in a tumor-specific phylogenetic approach to profile the ctDNA | Through blinded profiling of post-operative plasma, we observe evidence of adjuvant chemotherapy resistance and identify pts who are very likely to experience recurrence. | Phylogenetic ctDNA profiling tracks the subclonal nature of lung cancer relapse and metastases, providing a new approach for ctDNA-driven therapeutic studies |
| Remon *JCO Precis Oncol* 2019^11^ | 214 treatment-naive and previously treated pts with advanced NSCLC, of whom 156 were treatment-naive and 111 of these had successful analysis of tissue molecular profile (Amplicon-based NGS sequencing tests x 37 genes) | Assess feasibility and utility of ctDNA by amplicon-based NGS in pts with NSCLC | Concordance agreement, for those who had both ctDNA and tumor sampled, was 95%, with 81% sensitivity and 97% specificity; ctDNA detected actionable mutations in 17% of patients with no tissue available | Amplicon-based NGS was shown to be accurate and reliable in using ctDNA mutation testing to detect actionable mutations in NSCLC |
| Nabet *Cancer Res* 2020^12^ | 99 NSCLC pts receiving ICIs (Multi-analyte assay) | Show if ctDNA and circulating immune profiles are independently associated with durable clinical benefit | The DIREct-On assay predicted durable clinical benefit in the patient population and was validated by two independent cohorts | Combining ctDNA and profiling of circulating immune cells can provide accurate, noninvasive forecasting for NSCLC pts |
| Goldberg *Clin Cancer Res* 2018^13^ | 49 pts with mNSCLC receiving immunotherapy with an anti-PD-1 or anti-PD-L1 drug, 28 of whom had somatic mutations identified in plasma  (NGS hybrid-capture sequencing tests x 174 genes) | Assess whether predictions could be made on immunotherapy effectiveness based on changes to levels of ctDNA | Agreement between responses of ctDNA and radiographic testing, and response time was faster for ctDNA pts | A reduction in ctDNA level could be an early marker of treatment efficacy and survival in those being treated with ICIs |
| Anagnostou *Cancer Res* 2019^14^ | 38 pts with NSCLC, including 24 pts with mNSCLC being treated with ICIs as SoC or in a clinical trial and another cohort of 14 pts with stage I to IIIA surgically resectable NSCLC receiving anti-PD1 therapy (NGS hybrid-capture sequencing tests x 174 genes) | Assess whether ctDNA and change in T-cells could reflect therapeutic outcome for those NSCLC pts being treated with ICIs | Pts who responded to therapy had a complete reduction in ctDNA levels compared with non-responders; those who acquired resistance experienced a drop followed by another increase in ctDNA | Detecting ctDNA and T-cell changes can be used to inform immune targeted therapy treatment for pts with NSCLC |
| Hellmann *Clin Cancer Res* 2020^15^ | 31 pts with stage IV NSCLC being treated with PD-L1 blockade, with tumor tissue available for 24 pts  (NGS hybrid-capture sequencing tests x 174 genes) | ORR as assessed by investigators, confirmed by repeat imaging | All pts had detectable ctDNA prior to initiation of therapy, whereas 26.7 months after, 27 pts had undetectable ctDNA; 4 pts had detectable ctDNA and eventually progressed | Analyzing ctDNA is a noninvasive method to identify MRD in pts with long-term PD-L1 response and could be used to predict risk of progression |
| Aggarwal *JAMA Oncol* 2019^16^ | 323 pts with NSCLC, of whom 229 had concurrent plasma and tissue NGS (NGS hybrid-capture test x 74 genes) | Determine whether plasma NGS testing correlated to improved detection of mutations leading to better delivery of personalized therapies, by measuring the number of pts with targetable mutations detected in plasma vs. tissue NGS | In those pts with concurrent plasma and tissue NGS, actionable mutations were increased from 47 pts to 82 pts and 36 pts receiving a personalized therapy as a result of plasma NGS achieved complete response, partial response or stable disease | Using plasma NGS testing in routine management of stage IV NSCLC could increase detection of actionable mutations and improve application of targeted therapies |
| Paik *N Engl J Med* 2020^17^ | 152 pts with advanced or mNSCLC who had confirmed *MET* exon 14 skipping mutation, 99 of whom were followed for at least 9 months (*MET* exon 14 NGS hybrid-capture test x 74 genes) | ORR by independent review in pts who underwent at least 9 months follow-up | Response rate was 48% among 66 pts in the liquid-biopsy group compared to 50% among 60 pts in the tissue-biopsy group; 27 pts had positive results according to both | The use of tepotinib was associated with a partial response in half of pts with advanced NSCLC with *MET* exon 14 skipping mutation |
| Park *Cancer Res Treat* 2021^18^ | 39 pts with mNSCLC (*EGFR* RT-PCR) | Investigator confirmed ORR | Sensitivity of ctDNA at detecting the activating *EGFR* mutations was 74% for both methods (Mutyper and cobas^®^) | Osimertinib was effective as a first-line treatment of mNSCLC with activating *EGFR* mutations as detected by ctDNA and tissue testing |
| Denis *J Thorac Dis* 2019^19^ | 126 patients evaluated for *EGFR* mutations (*EGFR* RT-PCR) | *EGFR* mutation status concordance between matched tumor and plasma samples | Mutation status concordance between 126 matched patient samples was 96.0% | Data confirm ctDNA as an alternative sample for *EGFR* mutation analysis in patients with advanced NSCLC |
| **Trials supporting ctDNA LB testing during disease progression** | | | | |
| Horn *J Thorac Oncol* 2019^20^ | 76 pts with *ALK*+ NSCLC who were *ALK* TKI-naïve or had received *ALK* TKIs in the past (*ALK* NGS hybrid-capture test x 16 genes) | Assess feasibility of ctDNA NGS at identifying genomic mutations and monitor resistance response to treatment | Concordance of *ALK* fusion between LB and TB was 91% and genetic alterations were detected in 74% of pts | Identification of an *ALK*+ NSCLC subgroup who could respond better to treatment by ctDNA analysis demonstrates clinical utility |
| Giroux Leprieur *Oncoimmunology* 2018^21^ | 15 pts with advanced NSCLC treated with nivolumab (Large panel NGS) | Describe the predictive value of ctDNA on the efficacy of nivolumab in advanced NSCLC | ROC curve analyses showed good diagnostic performances for tumor response and clinical benefit, both for ctDNA  concentration at the first tumor evaluation (tumor response PPV: 100.0%; NPV: 71.0%; clinical benefit PPV: 83.3%; NPV 77.8%). Patients without ctDNA concentration increase >9% at 2 months had a long-term benefit of nivolumab | NGS analysis of ctDNA allows the early detection of tumor response and long-term clinical benefit with nivolumab in NSCLC |
| Dietz *EBioMedicine* 2020^22^ | 73 pts with *ALK*+ mNSCLC previously treated with TKIs (*ALK* RNA-based NGS test x 70 genes) | Assess the feasibility of combining of cfDNA assays such as sWGS with NGS to improve disease monitoring in *ALK*+ NSCLC | cfDNA mutations were detected in 58% of the pts sampled, some of which were actionable or druggable. Increases in ctDNA correlated to high-risk biomarkers and shorter pt survival | Combining these profiling methods could identify treatment failure and detect genomic alterations acquired during the course of treatment |
| Lamy *Cancers (Basel)* 2020^23^ | 137 NSCLC pts (*EGFR* RT-PCR) | Compare detection of variants in NSCLC pts between the already established cobas^®^ test and UltraSEEK Lung Panel on the MassARRAY | The cobas^®^ test detected a higher proportion of *EGFR* exon19 deletions and L858R mutations. UltraSEEK detected more T790M mutations | The UltraSEEK Lung Panel on the MassARRAY System is accurate at detecting ccfDNA and overall concordance between this method and cobas^®^ was 86% |
| Papadimitrakopoulou *Cancer* 2020^24^ | 1,036 pts with tissue-confirmed T790M+ advanced NSCLC with disease progression on first-line *EGFR* TKI therapy, 891 of whom were included for analysis (*EGFR* RT-PCR; ddPCR; NGS hybrid-capture test x 74 genes) | PFS determined by investigator assessment | Plasma testing was compared to cobas^®^ tissue testing and all 3 methods were found to have over 50% concordance (51, 58 and 66%), and detection of plasma T790M was associated with a larger baseline tumor and extrathoracic disease | All 3 testing methods used were deemed suitable for the clinic; in those patients with tissue T790M+ NSCLC, the absence of plasma T790M was associated with a longer PFS |

*ALK*, anaplastic lymphoma kinase; cfDNA, circulating free DNA; ccfDNA, circulating, cell-free DNA; ctDNA, circulating tumor DNA; *EGFR*, epidermal growth factor receptor; ddPCR, digital droplet PCR; ICI, immune checkpoint inhibitor; LB, liquid biopsy; *MET*, N-methyl-N′-nitroso-guanidine human osteosarcoma transforming gene; mNSCLC, metastatic NSCLC; MRD, minimal residual disease; NGS, next-generation sequencing; NPV, negative predictive value; NSCLC, non-small cell lung cancer; PCR, polymerase chain reaction; PD1, programmed cell death protein 1; PD-L1, programmed death-ligand 1; PPV, positive predictive value; PFS, progression free survival; pts, patients; ORR, objective response rate; ROC, receiver operating characteristic curve; RNA, ribonucleic acid; RT-PCR, real-time PCR; SoC, standard of care; sWGS, shallow whole genome sequencing; TB, tissue biopsy; TKI, tyrosine kinase inhibitor.

**REFERENCES**

1. Foundation Medicine Inc. FoundationOne® Liquid CDx Technical Information. <https://info.foundationmedicine.com/hubfs/FMI%20Labels/FoundationOne_Liquid_CDx_Label_Technical_Info.pdf>; Accessed July 1 2021.

2. Zehir A, Benayed R, Shah RH, et al. Mutational landscape of metastatic cancer revealed from prospective clinical sequencing of 10,000 patients. *Nature medicine* 2017;23:703-713.

3. Lee Y, Clark EW, Milan MSD, et al. Turnaround time of plasma next-generation sequencing in thoracic oncology patients: a quality improvement analysis. *JCO Precis Oncol* 2020;4:PO.20.00121.

4. Ferguson E. The strengths and weaknesses of whole-genome sequencing. *INSPIRE Student Health Sciences Research Journal* 2020;Autumn 2020:<https://inspirestudentjournal.co.uk/wp-content/uploads/2020/2010/Inspire-Student-Journal-Emily-Ferguson.pdf> (Accessed July 2021, 2011).

5. Mayo Clinic Laboratories, Mayo Foundation for Medical Education and Research. Whole Exome Sequencing (WES): Questions and Answers for Providers. 2018. <https://www.mayocliniclabs.com/it-mmfiles/Whole_Exome_Sequencing__WES_-_Questions_and_Answers_for_Providers.pdf>; Accessed July 1 2021.

6. Herbreteau G, Vallée A, Charpentier S, et al. Circulating free tumor DNA in non-small cell lung cancer (NSCLC): clinical application and future perspectives. *J Thorac Dis* 2019;11:S113-S126.

7. Gadgeel SM, Mok TSK, Peters S, et al. Phase II/III blood-first assay screening trial (BFAST) in treatment-naïve NSCLC: initial results from the ALK+ cohort. *Ann Oncol* 2019;30:Abstract LBA81_PR.

8. Leighl NB, Page RD, Raymond VM, et al. Clinical utility of comprehensive cell-free DNA analysis to identify genomic biomarkers in patients with newly diagnosed metastatic non-small cell lung cancer. *Clinical cancer research : an official journal of the American Association for Cancer Research* 2019;25:4691-4700.

9. Schrock AB, Zhu VW, Hsieh WS, et al. Receptor tyrosine kinase fusions and BRAF kinase fusions are rare but actionable resistance mechanisms to EGFR tyrosine kinase inhibitors. *J Thorac Oncol* 2018;13:1312-1323.

10. Abbosh C, Birkbak NJ, Wilson GA, et al. Phylogenetic ctDNA analysis depicts early-stage lung cancer evolution. *Nature* 2017;545:446-451.

11. Remon J, Lacroix L, Jovelet C, et al. Real-world utility of an amplicon-based next-generation sequencing liquid biopsy for broad molecular profiling in patients with advanced non-small-cell lung cancer. *JCO Precis Oncol* 2019;3:PO.18.00211.

12. Nabet BY, Esfahani MS, Hamilton EG, et al. A noninvasive approach for early prediction of therapeutic benefit from immune checkpoint inhibition for lung cancer. *Cancer Res* 2020;80:Abstract 5666.

13. Goldberg SB, Narayan A, Kole AJ, et al. Early assessment of lung cancer immunotherapy response via circulating tumor DNA. *Clinical cancer research : an official journal of the American Association for Cancer Research* 2018;24:1872-1880.

14. Anagnostou V, Forde PM, White JR, et al. Dynamics of tumor and immune responses during immune checkpoint blockade in non–small cell lung cancer. *Cancer Res* 2019;79:1214-1225.

15. Hellmann MD, Nabet BY, Rizvi H, et al. Circulating tumor DNA analysis to assess risk of progression after long-term response to PD-(L)1 blockade in NSCLC. *Clinical cancer research : an official journal of the American Association for Cancer Research* 2020;26:2849-2858.

16. Aggarwal C, Thompson JC, Black TA, et al. Clinical implications of plasma-based genotyping with the delivery of personalized therapy in metastatic non-small cell lung cancer. *JAMA oncology* 2019;5:173-180.

17. Paik PK, Felip E, Veillon R, et al. Tepotinib in non-small-cell lung cancer with MET exon 14 skipping mutations. *New England Journal of Medicine* 2020;383:931-943.

18. Park CK, Cho HJ, Choi YD, et al. A phase II trial of osimertinib as the first-line treatment of non-small cell lung cancer harboring activating EGFR mutations in circulating tumor DNA: LiquidLung-O-Cohort 1. *Cancer Research and Treatment* 2021;53:93-103.

19. Denis MG, Lafourcade MP, Le Garff G, et al. Circulating free tumor-derived DNA to detect EGFR mutations in patients with advanced NSCLC: French subset analysis of the ASSESS study. *J Thorac Dis* 2019;11:1370-1378.

20. Horn L, Whisenant JG, Wakelee H, et al. Monitoring therapeutic response and resistance: analysis of circulating tumor DNA in patients with ALK+ lung cancer. *J Thorac Oncol* 2019;14:1901-1911.

21. Giroux Leprieur E, Herbretau G, Dumenil C, et al. Circulating tumor DNA evaluated by next-generation sequencing is predictive of tumor response and prolonged clinical benefit with nivolumab in advanced non-small cell lung cancer. *Oncoimmunology* 2018;7:e1424675.

22. Dietz S, Christopoulos P, Yuan Z, et al. Longitudinal therapy monitoring of ALK-positive lung cancer by combined copy number and targeted mutation profiling of cell-free DNA. *EBioMedicine* 2020;62:103103.

23. Lamy PJ, van der Leest P, Lozano N, et al. Mass spectrometry as a highly sensitive method for specific circulating tumor DNA analysis in nsclc: a comparison study. *Cancers* 2020;12:3002.

24. Papadimitrakopoulou VA, Han JY, Ahn MJ, et al. Epidermal growth factor receptor mutation analysis in tissue and plasma from the AURA3 trial: Osimertinib versus platinum-pemetrexed for T790M mutation-positive advanced non-small cell lung cancer. *Cancer* 2020;126:373-380.
